# GPD1L-A306del modifies sodium current in a family carrying the dysfunctional SCN5A-G1661R mutation associated with Brugada syndrome

**DOI:** 10.1007/s00424-023-02882-0

**Published:** 2023-12-01

**Authors:** Francesca Semino, Fabrice F. Darche, Claus Bruehl, Michael Koenen, Heyko Skladny, Hugo A. Katus, Norbert Frey, Andreas Draguhn, Patrick A. Schweizer

**Affiliations:** 1 Department of Cardiology, Medical University Hospital Heidelberg, Heidelberg, Germany; 2 Institute of Physiology and Pathophysiology, Heidelberg University, Heidelberg, Germany; 3 Department of Molecular Neurobiology, Max Planck Institute for Medical Research, Heidelberg, Germany; 4 SYNLAB MVZ Humangenetik Mannheim GmbH, Mannheim, Germany; 5 German Center for Cardiovascular Research (DZHK), Partner Site Heidelberg/Mannheim, Heidelberg, Germany

**Keywords:** Brugada syndrome, BrS, *GPD1L*, *SCN5A*, Nav1.5, Sodium channel

## Abstract

Loss-of-function variants of *SCN5A*, encoding the sodium channel alpha subunit Nav1.5 are associated with high phenotypic variability and multiple cardiac presentations, while underlying mechanisms are incompletely understood. Here we investigated a family with individuals affected by Brugada Syndrome (BrS) of different severity and aimed to unravel the underlying genetic and electrophysiological basis.

Next-generation sequencing was used to identify the genetic variants carried by family members. The index patient, who was severely affected by arrhythmogenic BrS, carried previously uncharacterized variants of Nav1.5 (SCN5A-G1661R) and glycerol-3-phosphate dehydrogenase-1-like protein (GPD1L-A306del) in a double heterozygous conformation. Family members exclusively carrying SCN5A-G1661R showed asymptomatic Brugada ECG patterns, while another patient solely carrying GPD1L-A306del lacked any clinical phenotype.

To assess functional mechanisms, Nav1.5 channels were transiently expressed in HEK-293 cells in the presence and absence of GPD1L. Whole-cell patch-clamp recordings revealed loss of sodium currents after homozygous expression of SCN5A-G1661R, and reduction of current amplitude to ~ 50% in cells transfected with equal amounts of wildtype and mutant Nav1.5. Co-expression of wildtype Nav1.5 and GPD1L showed a trend towards increased sodium current amplitudes and a hyperpolarizing shift in steady-state activation and -inactivation compared to sole *SCN5A* expression. Application of the GPD1L-A306del variant shifted steady-state activation to more hyperpolarized and inactivation to more depolarized potentials.

In conclusion, SCN5A-G1661R produces dysfunctional channels and associates with BrS. *SCN5A* mediated currents are modulated by co-expression of GDP1L and this interaction is altered by mutations in both proteins. Thus, additive genetic burden may aggravate disease severity, explaining higher arrhythmogenicity in double mutation carriers.

**Supplementary Information:**

The online version contains supplementary material available at 10.1007/s00424-023-02882-0.

## Introduction

Brugada syndrome (BrS) is a rare inherited arrhythmia syndrome characterized by typical ECG changes that accounts for 20% of sudden cardiac deaths (SCD) in patients with structurally normal hearts [1–3]. Clinical symptoms comprise malignant ventricular arrhythmias, such as ventricular tachycardia (VT) or fibrillation (VF) resulting in syncope and cardiac arrest. Furthermore, multiple forms of supraventricular tachycardias (atrial fibrillation and flutter, AV-nodal re-entry tachycardia) are associated with BrS [1]. Little is known why some individuals are only mildly affected or even asymptomatic, whereas others exhibit a highly arrhythmogenic clinical picture. The diagnosis of BrS is substantially based on the presence of a type I ECG pattern, either occurring spontaneously or induced by the application of provoking drugs (Ajmalin, Flecainide etc.) [4–6]. The underlying pathomechanisms remain incompletely understood, and no causal medical therapy is available to date. Therefore, patients at risk for malignant arrhythmias require the implantation of an implantable cardioverter-defibrillator (ICD) to be protected from life threatening events [4].

The genetic origin of BrS has been first described in 1998 with the identification of pathogenic *SCN5A* mutations in affected patients [7]. Loss-of-function variants in *SCN5A* are the only proven genetic cause of BrS and were identified in 20–30% of the diagnosed patients [8]. *SCN5A* encodes for the voltage-gated sodium channel Nav1.5 underlying the fast sodium influx of the early phase of the myocardial action potential [8]. Changes in the biophysical properties of the sodium channel have been described as important mechanisms of the disease, typically shifting steady-state activation to the depolarized direction, steady-state inactivation to the hyperpolarized direction or prolonging recovery from inactivation [7]. Other studies identified reduced surface expression of sodium channels as a key player in BrS pathophysiology [9]. Interestingly, loss-of-function of the cardiac sodium channel is associated with other cardiac disorders as well, such as sinus node disfunction (SND), conduction defects, inherited atrial fibrillation (AF) and dilated cardiomyopathy (DCM) [10–12]. Gain-of-function variants, by contrast, are linked to the long QT syndrome (LQTS) type 3 [13].

In addition to *SCN5A* around 40 genes implicated in cardiac electrophysiology haven been reported to contribute to BrS, while causality of variants in those genes has been proven for none of them [8]. One of these genes is *GPD1L*, encoding the glycerol-3-phosphate dehydrogenase-1-like protein, which shares 84% homology with the glycerol-3-phosphate dehydrogenase 1 (GPD1) [14]. Although its function is not fully understood, these structural similarities suggest an involvement of GPD1L in the NAD^+^/NADH dependent reverse redox reaction of dihydroxyacetone phosphate to glycerol 3-phosphate. Previous studies indicate that a *GPD1L* variant increases the NADH concentrations and reduces surface expression of Nav1.5 through activation of protein kinase C (PKC) and phosphorylation of the channel [15, 16]. Hence, it is not surprising that *GPD1L* variants have been associated with BrS, but its exact contribution to disease mechanisms is yet unresolved.

In this study, two previously uncharacterized variants in the *SCN5A* and *GPD1L* genes were identified in a German family with members affected by BrS. By analyzing the functional effects of these variants alone or in combination, we aimed to explore the arrhythmogenic pathomechanisms of our patients and open new perspectives on the role of GPD1L as a sodium channel interactor and clinical modifier of BrS.

## Material and methods

### Clinical evaluation

The subjects investigated in this study included an index patient and three additional family members. The clinical evaluation started with a detailed medical history, and a family pedigree was drawn. A physical examination, a 12-lead resting electrocardiogram (ECG), exercise testing and 24 h Holter ECG recording as well as echocardiography and/or cardiac magnetic resonance imaging (MRI) were performed. Arrhythmogenicity of the index patient was evaluated by an electrophysiological investigation and programmed right ventricular stimulation. The father of the index patient was examined by Ajmaline challenge to assess inducibility of a type I Brugada pattern in the ECG, according to the recommendations of the European Society of Cardiology [4, 5]. All patients included in this study gave written informed consent for clinical and genetic investigation according to the research protocol, which has been approved by the local ethics committee. The investigations conform to the principles outlined in the Declaration of Helsinki.

### Genetic investigations

For genetic testing peripheral venous blood was drawn from each family member. All family members received routine clinical genetic counseling and were genetically tested using a next-generation sequencing (NGS) based BrS genetic panel covering 11 disease gene exon sequences (*SCN5A, SCN1B, SCN2B, SCN3B, CACNA1C, CACNB2, GPD1L, HCN4, KCNE3, KCND3, KCNJ8*) (Synlab Analytics & Services Germany GmbH, Mannheim, Germany).

### Plasmid construction

Wildytpe (WT) human heart *SCN5A* cDNA (NM_198056.3) was cloned into a pCMV directed expression vector and the *GPD1L* cDNA (NM_015141) cloned into the pCMV6-XL4 expression vector was obtained from OriGene Technologies (Rockville, MD 20850, US). To introduce the two mutations a site-directed mutagenesis was performed using the QuikChange Multi Site-Directed Mutagenesis Kit (Agilent Technologies, Santa Clara, California, USA). The sequences were then controlled via Supreme Sanger Sequencing (Eurofins Genomics, Luxembourg). To identify the transfected cells and control the efficiency of the transfection a peGFP-N1 plasmid was used. The plasmid was kindly provided by Dr. Julian Schröter (Division of Pediatric Epileptology, Center for Pediatric and Adolescent Medicine, Heidelberg University Hospital).

### Cell culture and transfection

HEK-293 cells (RRID:CVCL_0045) were cultivated in 100 × 20 mm Petri-dishes containing 10 ml of Dulbecco’s Modified Eagles Medium (DMEM, high glucose, GlutaMAX™ Supplement) supplemented with 10% fetal bovine serum (FBS), 1% Minimum Essential Medium Non-Essential Amino Acids (MEM NEAA 100X), 1% penicillin/streptomycin and 0.001% 2-Mercaptoethanol 50 mM (Gibco™, Thermo Fisher Scientific, Waltham, Massachusetts, USA) and incubated at 37 °C in a 5% CO_2_ incubator. For electrophysiological recordings 48 h prior to transfection cells were plated on Poly-L-lysine (Sigma-Aldrich, St. Louis, Missouri, USA) coated coverslips in a concentration of 2 × 10^3^ cells / 500 µl supplemented medium / coverslip (according to [17]). Transfection was performed with the Lipofectamine 3000 Transfection Kit (Invitrogen, Thermo Fisher Scientific). A total amount of 275 ng plasmid and 1.62 µl Lipofectamine 3000 per coverslip were used, independent of the number of different plasmids. The total amount of plasmids contained 20% peGFP-N1 and the remaining quantity contained different combinations of the previously described WT and mutant plasmids. Note that, with this protocol, the amount of *SCN5A* WT cDNA was fourfold higher compared to co-transfection with mutant and wildtype DNA of *SCN5A* and *GPD1L*. Recordings were performed on GFP expressing cells 3–4 days after transfection.

### Electrophysiology experiments

Whole-cell patch clamp experiments were performed at room temperature using an Axopatch 200B amplifier (Molecular Devices Corporation, Sunnyvale, CA, USA) and recorded with Signal Software (Version 4.11, CED, Cambridge, UK) using custom-made protocols. The bath solution contained 140 mM NaCl, 3 mM KCl, 1 mM MgCl_2_, 5 mM CaCl_2_, 10 mM HEPES and 20 mM Glucose (pH 7.35 adjusted with NaOH), while the pipette solution contained 120 mM CsCl, 10 mM NaCl, 10 mM HEPES, 10 mM EGTA, 10 mM Glucose, 2 mM MgATP and 0.1 mM NaGTP (pH 7.3 adjusted with KOH). The patch electrodes had a resistance between 2–3.5 MΩ. After reaching the whole-cell configuration cells were held at -80 mV for 5 min prior to recording. Capacitive transients were manually compensated, and the cell capacitance was documented by direct read-out from the respective dials. Series resistance was compensated for 60–80%. Measured currents were filtered at 5 kHz. Each recording session started with WT transfected cells to assure the functionality of the setup. Four different stimulation protocols were applied to assess different properties of the Nav1.5 channels. Protocols started with a 500 ms hyperpolarizing pulse at -120 mV to recover all channels from inactivation. For steady-state activation a depolarizing pulse of 20 ms was applied at different test potentials reaching from -70 to 45 mV (increment of 5 mV). For steady-state inactivation, a two-pulse protocol was used, starting with a depolarizing pulse of 500 ms to potentials between -120 and -10 mV (increment of 5 mV), followed by a test pulse to -10 mV for 20 ms. Recovery from fast and intermediate inactivation was evaluated with a two-pulse protocol. First, cells were depolarized to -10 mV either for 20 ms or for 1000 ms (P1). Then, cells recovered at -70 mV for 1–1024 ms (fast inactivation) or 1–1460 ms (intermediate inactivation), followed by a second 20 ms long pulse to -10 mV (P2). Tests were separated by an interpulse interval of 5 s at a holding potential of -70 mV. After completing the measurements, we controlled the offset potential of the open pipette. Only cells with an offset lower than ± 0.5 mV were accepted. Analysis was performed using Signal Software (Version 4.11, CED, Cambridge, UK) and Excel 365 (Microsoft, Redmond, Washington, USA). First, the leak current of each cell, measured between -65 and -75 mV, was subtracted. Cells were excluded from the analysis if the holding current (at -80 mV) was more negative than -200 pA or the measurements were unstable. Then, the half maximal potential (Vh,a), the slope factor (Vc,a) of steady-state activation and the permeability g_max_ of the cells were calculated using a combination of the Goldman-Hodgkin-Katz equation and Boltzmann equation (1) [18]. Current density was determined by dividing the cell current by the cell capacitance.

1 $$\Delta \mathrm{I}\left(\mathrm{V}\right)=V*\frac{{\mathrm{g}}\mathrm{max }}{1+\mathrm{exp}\left(\frac{Vh,a -V}{Vc,a}\right)}*\frac{\left(\left[{\mathrm{Nain}}\right]\mathrm{/}\left[{\mathrm{Naout}}\right]\right)-\mathrm{exp}\left(-\alpha V\right)}{1-\mathrm{exp}\left(-\alpha V\right)}, \alpha =\frac{F}{R*T}$$

I: sodium current
V: potential
*g*_*max*_: max. permeability
V_h,a_: half maximal potential of activation
V_c,a_: slope factor (at V_h,a_)
Na_in_: sodium concentration in the intracellular solution
Na_out_: sodium concentration in the extracellular solution
F: Faraday constant
R: gas constant
T: absolute room temperature

The half maximal potential (Vh,i) and the slope factor (Vc,i) of steady-state inactivation were determined using a Boltzmann Eq. (2) in the form:

2 $$\frac{\mathrm{I}\left(\mathrm{V}\right)}{\mathrm{Imax}}=1-\frac{{1} \, }{1+\mathrm{exp}(\frac{Vh,i -V}{Vc,i})}$$

I: sodium current
V: potential
V_h,i_: half maximal potential of inactivation
V_c,i_: slope factor

The recovery properties were best fitted with a single exponential function (3) and the time constant τ for the fast (τf) and intermediate (τi) inactivation were calculated.

3 $$\frac{\mathrm{P}2}{\mathrm{P}1}=\mathrm{A}+(1-(\mathrm{A}1*\mathrm{exp}(-\frac{\mathrm{t}}{\uptau })$$

P2: second pulse
P1: first pulse
A: amplitude
t: time
τ: time constant

### Statistical analysis

Statistical analysis was performed with GraphPad Prism (Version 9.2.0, GraphPad Software, RRID:SCR_002798, San Diego, California, USA). Results are shown as mean ± standard error of the mean (SEM). Cells were excluded from the analysis if their values exceeded three standard deviations (mean ± 3*SD). For statistical comparison, data distributions were first examined with the D'Agostino and Pearson normality test. Normally distributed data were then compared either with the unpaired Student *t* test with Welch’s correction or with ANOVA with Bonferroni’s correction. The Mann Whitney Tests and the Kruskal Wallis Tests with Dunn’s comparison were used for non-parametric data. Results were considered statistically significant with p < 0.05.

## Results

### Clinical characteristics of the patients

**Fig. 1:**
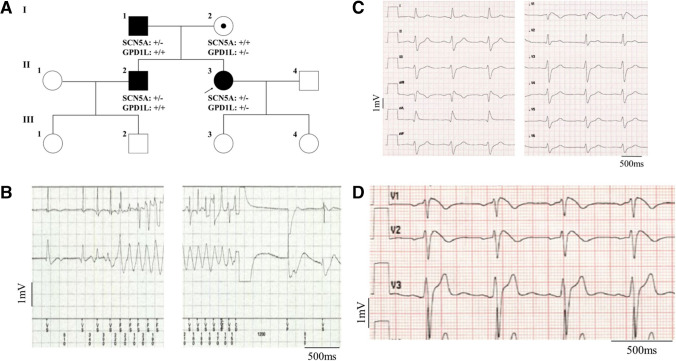
Affected members and ECG abnormalities in the BrS family. (**A**) Pedigree of the family. Filled symbols indicate BrS affected individuals, pointed symbols denote gene carrying individuals and open symbols unaffected family members. The genotype of each individual, if known, is reported on the bottom: + / + indicates WT, ± heterozygous and -/- homozygous genotype. (**B**) Ventricular fibrillation episode with ICD shock in individual II.3. (**C**) Spontaneous type I BrS ECG of individual II.3. (**D**) Spontaneous type I BrS ECG of individual II.2

The 41-year-old index patient (Fig. 1A, individual II.3) was referred to our hospital with recurrent pre-syncope, palpitations, and ECG changes suggestive of Brugada syndrome. At clinical admission 12-channel resting ECG exhibited a spontaneous type 1 Brugada pattern and a bifascicular conduction block (right bundle branch block and left anterior hemiblock). Holter recording revealed polymorphic premature ventricular contractions (PVC) and non-sustained VT, while echocardiography and cardiac MRI excluded structural heart disease as a possible arrhythmogenic substrate. To further examine conduction velocity and inducibility of tachyarrhythmias an electrophysiology study was performed. Intracardiac ECG revealed prolonged infra-Hisian conduction (HV = 76 ms), but dual side programmed right ventricular stimulation did not elicit ventricular tachycardia. Nevertheless, due to the recurrent pre-syncope and documented ventricular arrhythmias a single-chamber ICD was implanted. Five months after ICD implantation VF occurred at rest and was terminated by ICD shock delivery (Fig. 1B). Furthermore, recurrent atrial tachycardias caused ongoing symptoms but could not adequately be treated by catheter ablation, due to their instable and polymorphic character. Thus, quinidine was administered orally, and the patient was free from sustained atrial or ventricular tachyarrhythmias since then.

Subsequently, the father (I.1), mother (I.2) and brother (II.2) of the index patient were examined (Table 1). Her father (I.1) did not present any specific cardiac symptoms but reported a syncope at the age of 60 years in the course of a common cold and had a history of irritable bowel syndrome in the adolescence. An echocardiography excluded structural heart disease. The ECG showed incomplete right bundle brunch block and Ajmaline challenge induced a type I Brugada pattern (Fig. 1C). The mother of the index patient (I.2) did not report any cardiac disorder or complaint and revealed a normal ECG. The brother of the index patient (II.2) presented a spontaneous type I Brugada pattern in the resting ECG (Fig. 1D) but denied syncope, palpitations, or any symptoms suggestive of BrS. Noteworthy, Crohn’s disease was diagnosed many years ago and he was treated with anti-inflammatory therapy.

**Table 1:** Clinical and genetical characteristics of variants carrying individuals

Individual	Sex	Current age (yr)	Genotype	BrS phenotype (age at diagnose in yr)	BrS ECG	Symptoms (age in yr)	Cardiac conduction diseases (age at diagnose in yr)	Structural cardiac abnormalities (imaging technique)	Therapy (age in yr)	Other diagnoses
I.1	m	79	SCN5A c.4981G > A, p. G1661R	Affected (76)	Inducible type I ECG	Syncope (60)	/	None (echocardiography)	/	Irritable bowel syndrome
I.2	f	76	GPD1L c.917_919del, p. A306del	Not affected	/	/	/	/	/	/
II.2	m	43	SCN5A c.4981G > A, p. G1661R	Affected (43)	Spontaneous type I ECG	/	/	None (echocardiography)	/	Crohn’s disease
II.3	f	41	SCN5A c.4981G > A, p. G1661R GPD1L c.917_919del, p.A306del	Severely affected (37)	Spontaneous type I ECG	Syncope (32 and 37)	bifascicular conduction block (36), polymorphic PVC and non-sustained VT (36), prolonged infra-Hisian conduction (36), VF with ICD shock (37), recurrent multifocal AT (37)	None (cardiac-MRI, echocardiography)	ICD-implantation (37), Quinidine 600 mg/d (37)	Thyroid cancer

*m* = male; *f* = female; *ICD* = implantable cardioverter-defibrillator; *PVC* = premature ventricular contractions; *VT* = ventricular tachycardia; *VF* = ventricular fibrillation; *AT* = atrial tachycardia

### Genetic investigations

**Fig. 2:**
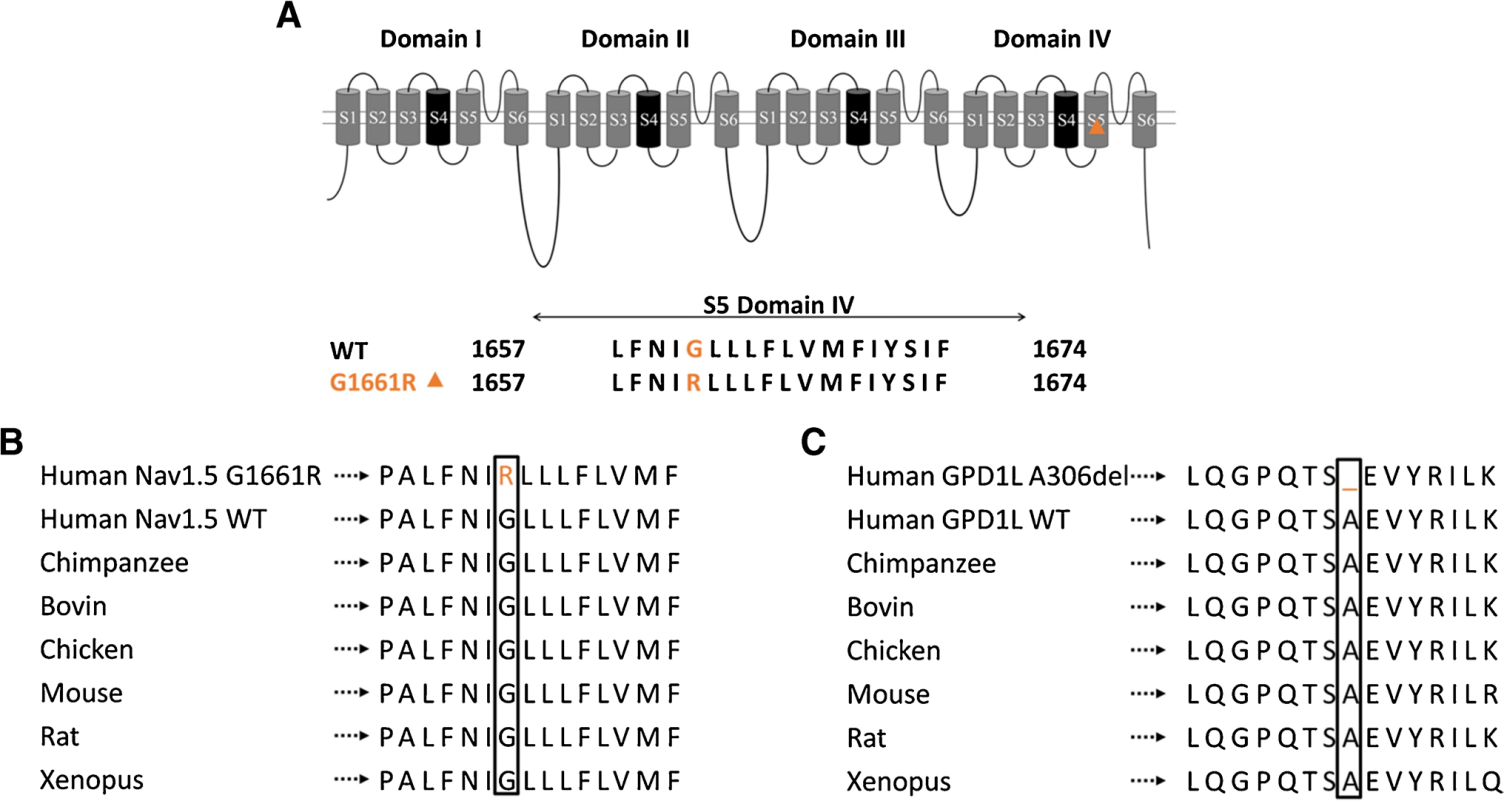
Both identified variants are localized in highly conserved regions of the respective proteins. (**A**) Scheme of the sodium channel and sequence of the subregion 5 of domain IV. The localization of the reported variant is symbolized with an orange triangle. (**B**—**C**) Alignment of parts of the SCN5A and GPD1L proteins from various species. The variants, written in orange, are located in highly conserved regions among the species

Genetic screening of the index patient was performed using a NGS based panel to examine genes associated with BrS. The analysis of exon sequences identified two previously uncharacterized heterozygous variants. The first variant *SCN5A* c.4981G > A (p. G1661R) causes a mutation in subdomain 5 of domain IV of the cardiac sodium channel Nav1.5 (Fig. 2A) which is part of the pore forming domain. The second variant *GPD1L* c.917_919del, (p. A306del) was identified in the glycerol-3-phosphate-dehydrogenase-1-like gene that has been reported to modulate cardiac sodium currents [14]. Both are rare genetic variants and are localized in highly conserved regions of the respective genes (Fig. 2B-C).

Mutation screening of the father (I.1) of the index patient revealed that he carries the *SCN5A* c.4981G > A (p. G1661R) variant in a heterozygous state but not the *GDP1L* mutant. Likewise, the index patient’s brother (II.2) carried the *SCN5A* variant without changes in the *GPD1L* gene. The index patient’s mother (I.2), on the other hand, carried the *GPD1L* c.917_919del (p. A306del) variant in a heterozygous fashion, but not the *SCN5A* variant (Table 1).

### SCN5A-G1661R causes sodium channel “loss-of-function”

**Fig. 3:**
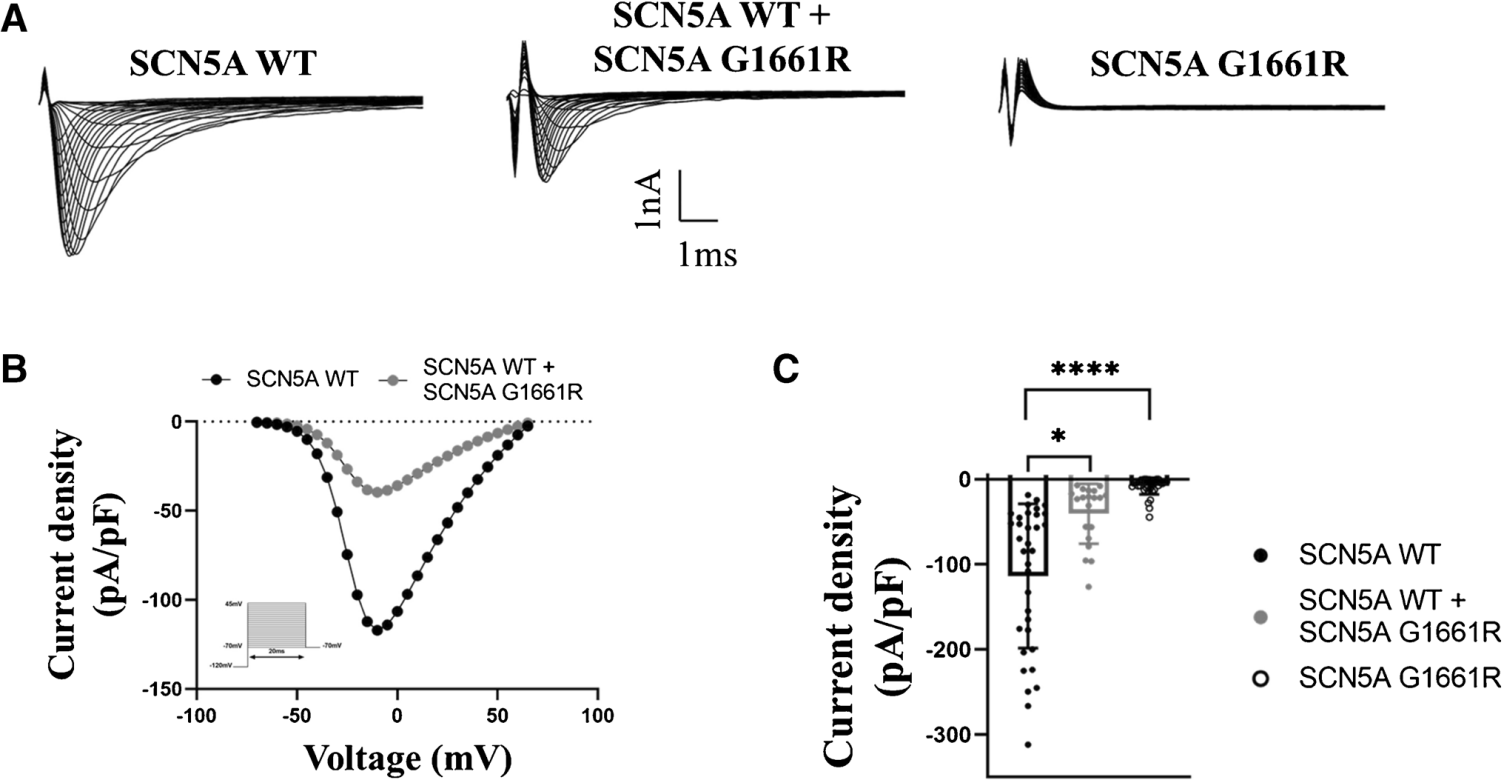
SCN5A-G1661R produces non-functional channels. (**A**) Representative current traces of cells transfected exclusively with the WT, the mutant or equivalent amounts of both *SCN5A* plasmids. (**B**) Current voltage relationship for WT (black circles) and *SCN5A* heterozygous (grey circles) transfected cells. The solid lines were determined using Eq. (1) with the averaged parameters. (**C**) Mean current density for each type of cell determined by normalizing the mean current at -10 mV to the mean cell capacitance. The mean cell capacitances were 11.5 ± 0.7 pF (SCN5A-WT), 13.1 ± 0.9 pF (SCN5A-WT + SCN5A-G1661R) and 11.9 ± 0.8 pF (SCN5A-G1661R), resp. ^*^p < 0.05, ^****^p < 0.0001 vs SCN5A-WT

To examine the influence of the SCN5A-G1661R variant on the sodium current, HEK**-**293 cells were transiently transfected with either WT, mutant or with equal amounts of both *SCN5A* plasmids, and currents were measured by whole-cell voltage clamp recordings. Current traces representative for each group are depicted in Fig. 3A. The mean current density was significantly reduced in the *SCN5A* variant as compared to the WT sodium current (Fig. 3B-C). Measurements at -10 mV showed a mean WT current density of -113.7 ± 14.5 pA/pF in cells transfected with wildtype *SCN5A*. Heterozygous transfection reduced the current density by 64.5% and homozygous transfection with mutant channels by 93.1% (-40.3 ± 7.9 pA/pF [p = 0.01] and -7.9 ± 1.6 pA/pF [p < 0.0001]), resp. (Fig. 3C). The biophysical properties of steady-state activation, inactivation and recovery from fast and intermediate inactivation did not differ between the groups (Table 2, Online Resource 1).

**Table 2:** Biophysical properties of the sodium current in HEK**-**293 cells of all measured transfection groups

Transfection group	Current density at -10 mV (pA/pF)	Vh,a (mV)	Vc,a	Vh,i (mV)	Vc,i
SCN5A-WT	n = 34 -113.7 ± 14.5	n = 34 -22.3 ± 1.0	n = 34 7.1 ± 0.4	n = 34 -79.1 ± 0.9	n = 34 5.2 ± 0.2
SCN5A-WT + SCN5A-G1661R	n = 20 -40.3 ± 7.9*	n = 22 -22.9 ± 1.5	n = 22 7.5 ± 0.5	n = 28 -78.7 ± 1.2	n = 28 6.4 ± 0.4**
SCN5A-G1661R	*n* = *38* -7.9 ± 1.6****	/	/	/	/
SCN5A-WT + GPD1L-WT	n = 26 -147.1 ± 16.5	n = 26 -25.3 ± 1.0*	n = 26 7.2 ± 0.3	n = 30 -86.4 ± 1.3****	*n* = *30* 6.9 ± 0.3****
SCN5A-WT + GPD1L-WT + GPD1L-A306del	n = 15 -128.3 ± 19.0	*n* = *15* -*28.6 ± 1.5*	*n* = *15* * 6.1 ± 0.5*	n = 16 -81.5 ± 1.0^†^	*n* = *16* * 6.1 ± 0.2*
SCN5A-WT + GPD1L-A306del	n = 13 -111.2 ± 16.7	n = 13 -32.2 ± 2.1^††^	n = 13 5.8 ± 0.6^†^	n = 14-85.2 ± 1.2	n = 14 6.4 ± 0.2
SCN5A-WT + SCN5A-G1661R + GPD1L-WT	*n* = *14* -*87.7 ± 10.2*^†^	n = 14 -23.3 ± 1.9	n = 14 8.0 ± 0.4	*n* = *17* *-84.7* ± *1.3*	*n* = *17* * 5.9 ± 0.1*
SCN5A-WT + SCN5A-G1661R + GPD1L-WT + GPD1L-A306del	n = 15 -98.2 ± 13.7^†^	n = 15 -28.2 ± 1.7	n = 15 6.1 ± 0.5 ‡	n = 13 * -83.0 ± 1.1*	n = 13 6.0 ± 0.2
SCN5A-WT + SCN5A-G1661R + GPD1L-A306del	n = 14 -87.4 ± 16.6^†^	*n* = *14* -*23.0 ± 1.5*	n = 14 7.3 ± 0.5	n = 13 * -80.8 ± 1.0*	n = 13 5.9 ± 0.2

Data are presented as mean ± SEM. *n* = number of measured cells

Data in italics were not distributed normally, according to the D'Agostino and Pearson normality test

^*^p < 0.05, ^**^p < 0.01, ^***^p < 0.001, ^****^p < 0.0001 vs SCN5A-WT

^†^p < 0.05, ^††^p < 0.01, ^†††^p < 0.001, ^††††^p < 0.0001 vs SCN5A-WT + GPD1L-WT

^‡^p < 0.05, ^‡‡^p < 0.01, ^‡‡‡^p < 0.001, ^‡‡‡‡^p < 0.0001 vs SCN5A-WT + SCN5A-G1661R + GPD1L-WT

### GPD1L wildtype alters the biophysical properties of sodium channels

**Fig. 4:**
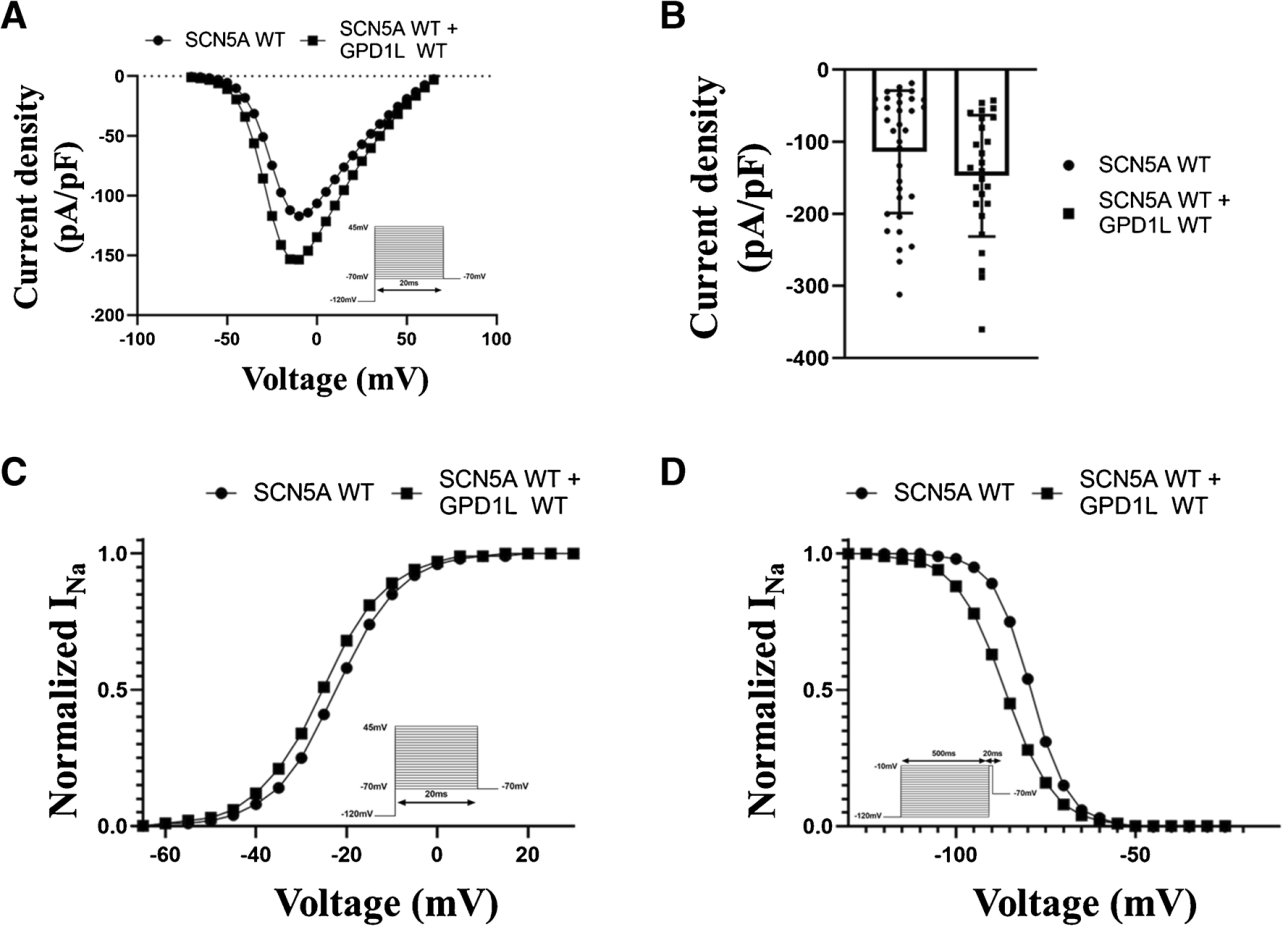
GPD1L changes the steady-state activation and inactivation properties of the sodium channels. (**A**) Current voltage relationship for cells transfected either exclusively with the WT *SCN5A* plasmid (black circles) or co-expressed with the WT *GPD1L* plasmid (black squares). The solid lines were determined using Eq. (1) with the averaged parameters. (**B**) Mean current density for each type of cell determined by normalizing the mean current at -10 mV to the mean cell capacitance. The mean cell capacitances were 11.5 ± 0.7 pF (SCN5A-WT) and 11.3 ± 0.7 pF (SCN5A-WT + GPD1L-WT), resp. (C-D) Voltage dependence of steady-state activation (**C**) and inactivation (**D**). The solid lines were determined using Eqs. (1) and (2) with the averaged parameters

To investigate the influence of the GPD1L protein on sodium current, cells transfected exclusively with the *SCN5A*-WT plasmid were compared with cells co-transfected with the *GPD1L*-WT plasmid. The current–voltage plot showed a trend towards an increased amplitude of voltage-activated sodium currents in co-transfected cells (Fig. 4A). At -10 mV cells expressing both plasmids showed a current density of -147.1 ± 16.5 pA/pF which is 29.4% higher than cells expressing *SCN5A*-WT alone (-113.7 ± 14.5 pA/pF [p = 0.14]) (Fig. 4B). Co-expression of GPD1L altered the steady-state activation and inactivation properties of Nav1.5 (Table 2). Both the half-maximal potential of steady-state activation and inactivation were significantly shifted towards hyperpolarization by ~ 3 mV (Vh,a = -22.3 ± 1.0 mV versus Vh,a = -25.3 ± 1.0 mV [p = 0.03]) and ~ 7 mV (Vh,i = -79.1 ± 0.9 mV versus Vh,i = -86.4 ± 1.3 mV [p < 0.0001]), in co-transfected cells (Fig. 4C-D). Further, while the slope factor of steady-state activation of co-transfected cells was comparable to that of *SCN5A*-WT (Vc,a = 7.1 ± 0.4 versus Vc,a = 7.2 ± 0.3 [p = 0.84], resp.), the slope factor of steady-state inactivation was steeper in the presence of *GDP1L* (Vc,i = 5.2 ± 0.2 versus Vc,i = 6.9 ± 0.3 [p < 0.0001], resp.). No changes were detected in recovery from fast and intermediate inactivation (Online Resource 2).

### Effects of GPD1L-A306del on sodium channel activation and inactivation properties 

**Fig. 5:**
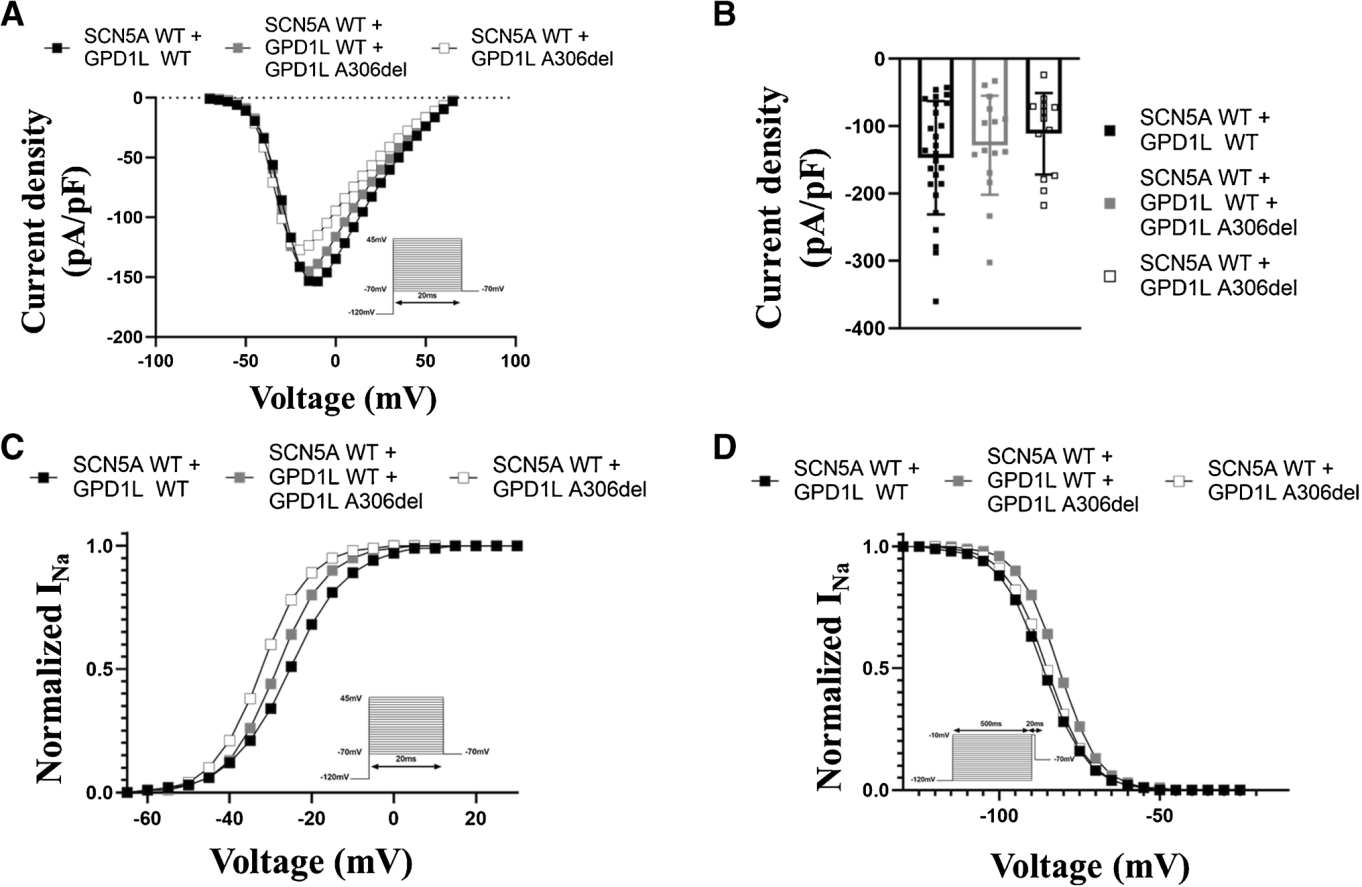
GPD1L-A306del shifts the steady-state activation of the sodium channels towards hyperpolarization, while altering the steady-state inactivation toward depolarization. (**A**) Current voltage relationship for cells transfected with the WT *SCN5A* plasmid co-expressed either with the *GPD1L* WT (black squares), mutant (white squares) or both plasmids (grey squares). The solid lines were determined using Eq. (1) with the averaged parameters. (**B**) Mean current density for each type of cell determined by normalizing the mean current at -10 mV to the mean cell capacitance. The mean cell capacitances were 11.3 ± 0.7 pF (SCN5A-WT + GPD1L-WT), 12.7 ± 0.6 pF (SCN5A-WT + GPD1L-WT + GPD1L-A306del) and 13.1 ± 0.5 pF (SCN5A-WT + GPD1L-A306del), resp. (**C**-**D**) Voltage dependence of steady-state activation (**C**) and inactivation (**D**). The solid lines were determined using Eqs. (1) and (2) with the averaged parameters

To analyze the impact of the newly identified GPD1L-A306del variant on sodium current, cells were co-transfected with *SCN5A*-WT and *GPD1L* plasmids in different configurations. In this paragraph, cells transfected with the *SCN5A*-WT and co-transfected with the *GPD1L*-WT plasmid will be referred to as WT, cells transfect with the *SCN5A*-WT and the mutant *GPD1L* plasmid will be referred to as homozygous, and cells transfected with *SCN5A*-WT and equivalent amount of both *GPD1L* plasmids will be referred to as heterozygous. The sodium current density for the heterozygous and homozygous groups showed a trend towards reduction compared to WT which was, however, not significant (-128.3 ± 19.0 pA/pF [heterozygous; p = 0.90] and -111.2 ± 16.7 pA/pF [homozygous; p = 0.34] versus -147.1 ± 16.5 pA/pF (WT), at -10 mV resp.) (Fig. 5A-B). Interestingly, the *GPD1L* variant shifted the steady-state activation and inactivation properties of the sodium channels in different directions (Table 2). The half-maximal potential of steady-state activation showed a trend towards hyperpolarization by ~ 3 mV in the heterozygous and a significant hyperpolarizing shift by ~ 7 mV in the homozygous conformation (Vh,a = -28.6 ± 1.5 mV [heterozygous; p = 0.15] and Vh,a = -32.2 ± 2.1 mV [homozygous; p = 0.003] versus Vh,a = -25.3 ± 1.0 mV (WT), resp.). The slope factor showed a trend to or was less steep in both groups (Vc,a = 6.1 ± 0.5 [heterozygous; p = 0.16] and Vc,a = 5.8 ± 0.6 [homozygous; p = 0.04] versus Vc,a = 7.2 ± 0.3 (WT), resp.) (Fig. 5C). Conversely, half-maximal potential of steady-state inactivation was shifted towards depolarization by ~ 5 mV in the heterozygous expression compared to WT (Vh,i = -81.5 ± 1.0 mV versus Vh,i = -86.4 ± 1.3 mV [p = 0.02], resp.). In this configuration the slope factor of steady-state inactivation was more steep than WT (Vc,i = 6.1 ± 0.2 versus Vc,i = 6.9 ± 0.3 [p = 0.09], resp.). Homozygous expression of the *GPD1L* variant did not alter steady-state inactivation properties compared to WT (Fig. 5D). No changes in the recovery properties from fast and intermediate inactivation were meassured (Online Resource 3).

### Co-expression of SCN5A-G1661R and GPD1L-A306del reduces sodium current density

**Fig. 6:**
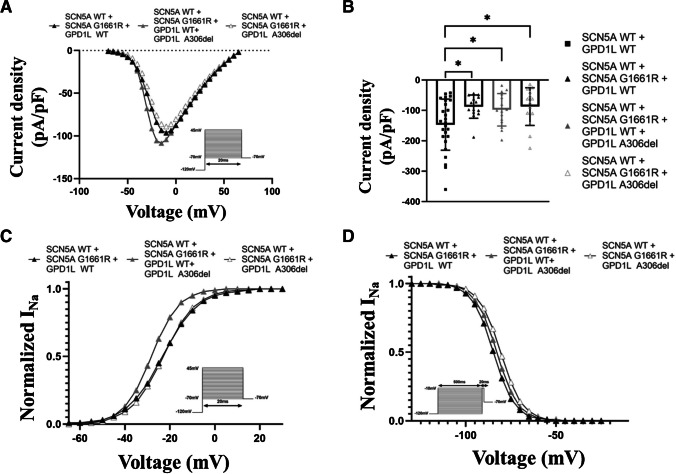
SCN5A-G1661R and GPD1L-A306del co-expression changes the steady-state activation and inactivation properties of the sodium channels, while reducing the sodium current. (**A**) Current voltage relationship for cells transfected with the WT and mutant *SCN5A* plasmid co-expressed either with the WT (black triangles), the mutant (white triangles) or both *GPD1L* plasmids (grey triangles). The solid lines were determined using Eq. (1) with the averaged parameters. (**B**) Mean current density for each type of cell determined by normalizing the mean current at -10 mV to the mean cell capacitance. The mean cell capacitances were 11.3 ± 0.7 pF (SCN5A-WT + GPD1L-WT), 12.8 ± 0.6 pF (SCN5A-WT + SCN5A-G1661R + GPD1L-WT), 14.7 ± 1.1 pF (SCN5A-WT + SCN5A-G1661R + GPD1L-WT + GPD1L-A306del) and 16.2 ± 1.2 pF (SCN5A-WT + SCN5A-G1661R + GPD1L-A306del), resp. (C-D) Voltage dependence of steady-state activation (**C**) and inactivation (**D**). The solid lines were determined using Eqs. (1) and (2) with the averaged parameters. ^*^p < 0.05 vs SCN5A-WT + GPD1L-WT

To elucidate possible interactions between the two variants, we next transfected cells with equal amounts of WT and mutant *SCN5A* plasmids and one of the following: *GPD1L-*WT plasmid (here referred to as *SCN5A* w/m + *GPD1L* w/w), mutant *GPD1L* plasmid (here referred to as *SCN5A* w/m + *GPD1L* m/m), or equivalent amounts of both *GPD1L* plasmids (here referred to as *SCN5A* w/m + *GPD1L* w/m). When comparing the current–voltage relationship of different groups, the *GPD1L* variant did not alter current densities significantly (Fig. 6A). However, the *SCN5A* variant had a significant impact on current density, as detected by comparing these cells with cells described in the previous paragraph presenting the *GPD1L* variant but not the *SCN5A* variant (Fig. 6B). In fact, at -10 mV, cells transfected with the *SCN5A* variant, regardless of co-transfection with *GPD1L* (either *SCN5A* w/m + *GPD1L* w/w, *SCN5A* w/m + *GPD1L* w/m, *SCN5A* w/m + *GPD1L* m/m), showed a marked reduction of current density compared with cells transfected with *SCN5A* and *GPD1L* WT (-87.7 ± 10.2 pA/pF *[p* = *0.03]* and -98.2 ± 13.7 pA/pF [p = 0.03] and -87.4 ± 16.6 pA/pF [p = 0.02] versus -147.1 ± 16.5 pA/pF, resp.). The influence of the variants was further analyzed by recording the steady-state activation and inactivation properties of sodium channels (Table 2). The half-maximal potential of steady-state activation showed again a trend towards hyperpolarization by ~ 5 mV in the *SCN5A* w/m + *GPD1L* w/m expression compared to *SCN5A* w/m + *GPD1L* w/w (Vh,a = -28.2 ± 1.7 mV versus Vh,a = -23.3 ± 1.9 mV [p = 0.08], resp.). The slope factor of steady-state activation was less steep in this group (Vc,a = 6.1 ± 0.5 versus Vc,a = 8.0 ± 0.4 [p = 0.01], resp.). The *SCN5A* w/m + *GPD1L* m/m cells, by contrast, displayed steady-state activation properties comparable to *SCN5A* w/m + *GPD1L* w/w (Fig. 6C). Conversely, the half-maximal potential of steady-state inactivation showed a trend towards depolarization by ~ 2 mV for the *SCN5A* w/m + *GPD1L* w/m and by ~ 4 mV for the *SCN5A* w/m + *GPD1L* m/m cells (Vh,i = -83.0 ± 1.1 mV [p > 0.99] and Vh,i = -80.8 ± 1.0 mV [p = 0.08] versus Vh,i = -84.7 ± 1.3 mV, resp.). The slope factor of steady-state inactivation was comparable for the three groups (Fig. 6D). Again, no changes in the recovery properties from fast and intermediate inactivation could be identified (Online Resource 4).

## Discussion

In this study we investigated a German family with individuals affected by BrS of different severity and explored the underlying pathomechanisms using panel-based genetic sequencing and patch-clamp recordings.

Two previously uncharacterized variants in genes associated with BrS, *SCN5A* and *GPD1L*, were identified in family members. Individuals I.1 and II.2, presenting only the heterozygous SCN5A-G1661R variant, showed an asymptomatic BrS phenotype, while individual I.2, heterozygously carrying the GPD1L-A306del variant, did not present any phenotype. The index patient (II.3), however, carried both variants and was affected by a highly arrhythmic course of BrS with additional signs of cardiac conduction disease.

We used whole-cell voltage-clamp recordings to explore functional mechanisms possibly underlying the effects of the variants detected in our patients. Our results showed that HEK-293 cells transfected with SCN5A-G1661R did not produce measurable sodium current. Co-expression experiments using WT and G1661R plasmids in equivalent concentrations, showed ~ 50% loss-of-current compared to the same amount of WT plasmid. We note that current densities are broadly distributed (see Fig. 3C, esp. for wildtype), probably reflecting different maturity stages of the continuously dividing cells at the time of transfection. With the strong CMV-promotor and the lipofectamine protocol used for transfection it is unlikely that differences in the number of plasmids taken up by individual cells played a major role for current amplitude variability. This is also underlined by the increase in current density upon co-transfection of *SCN5A*-WT and *GPD1L*-WT, compared to *SCN5A*-WT alone. This increase occurred despite the fact that, in co-expression experiments, the amount of *SCN5A* cDNA was 50% lower than in experiments solely using *SCN5A*. Despite the non-linear relationship between cDNA dose and functional expression levels, the systematic decrease in sodium current density for expression of SCN5A-WT, SCN5A-WT/SCN5A-G1661R and SCN5A-G1661R strongly suggests a non-functional mutant channel variant without dominant negative effect on the native sodium channels. Additionally, we cannot exclude that mutated Nav1.5 channels are expressed and inserted into the membrane, but incapable to function. Together, the results are consistent with current literature which associates loss-of-function *SCN5A* mutations with BrS [8].

Although single *SCN5A* variants may suffice to cause BrS in patients [7], cumulating data suggest that additive effects of variants in susceptibility genes may aggravate the clinical course of the disease [8]. The genetic predisposition in sum may determine why some individuals are only mildly affected or even asymptomatic, and others exhibit a highly arrhythmogenic clinical picture. This is the case in the family reported here with the index patient necessitating ICD implantation due to ventricular arrhythmias and requiring adequate ICD shock, while her relatives showed only mild phenotypes. In fact, although the SCN5A-G1661R variant may be sufficient to cause BrS in patients, a possible explanation for the aggravation of the clinical phenotype in the index patient was identified by NGS based screening providing a novel *GPD1L* gene variant (GPD1L-A306del) in the index patient.

At present, the function of GPD1L and its interaction with sodium channels are not fully understood. Due to its high homology to the GPD1 protein, a similar function has been assumed. GPD1 catalyzes the reversible redox reaction of dihydroxyacetone phosphate (DHAP) to glycerol-3-phosphate (G3P) using NADH/NAD + as the electron donor [14, 19, 20]. Studies on a *GPD1L* variant have supported this hypothesis, showing that the mutation causes an increment of NADH and G3P inside the cell, which then activates the calcium-dependent protein kinase C (PKC) [15, 16]. PKC is known to decrease single channel conductance and membrane expression of voltage-gated sodium channels, thereby reducing the sodium current [15, 16, 21, 22]. On the other hand, under normal conditions, a WT GPD1L mediated NAD^+^ dependent cascade activates protein kinase A (PKA) which increases the sodium current [15, 23, 24]. Our present findings are in line with these data, showing a trend towards increased sodium current in presence of GPD1L-WT and a trend to current reduction in presence of GPD1L-A306del. Nevertheless, there is no indication that GPD1L-A306del alone can cause BrS, as the patient solely carrying this variant (I.2), was completely asymptomatic. In addition to its known effects on sodium channel trafficking, our data reveal that GPD1L may modify Nav1.5 biophysical properties by shifting steady-state activation and inactivation towards hyperpolarized potentials. These effects have not been described previously, suggesting a new link between GPD1L and Nav1.5. Indeed, glutathione-S-transferase (GST)-pulldown assays indicate a close proximity of both proteins [16]. However, the precise localization of their interaction is currently not known [16] and an indirect influence of GPD1L on the sodium channel cannot be excluded. Such an effect may be, for example, mediated by PKA activation or by interaction with other sodium channel binding proteins. Involvement of PKA would be in line with the observed hyperpolarizing shift of steady-state activation and inactivation in our experiments, as similar alterations have been found after PKA activation [25]. Other proteins have been shown to alter the amplitude and gating properties of sodium currents. In ankyrin-B knock-out neonatal mice, for example, the sodium current density was reduced and a hyperpolarizing shift in the kinetics of Nav1.5 was observed [26]. Given these complex interactions, it is well feasible that the effects of GDPL1 and its mutants depend on the specific cellular environments. Thus, further studies of the direct and indirect mechanisms of sodium channel interaction with GPD1L are needed and should also be performed in native or iPSC-derived cardiac myocytes.

At present, only a few *GPD1L* variants have been identified in patients affected by BrS, sudden infant death syndrome, diabetic dead-in-bed syndrome, cardiac conduction disorder, atrial fibrillation and early repolarisation syndrome, but only 6 of them have been functionally characterized [14, 27–35]. Among these, P112L showed a hyperpolarizing shift in steady-state inactivation of sodium currents, P112L, A280V and R189X reduced the expression of GPD1L and/or Nav1.5 proteins, and all mutants were associated with a reduction of sodium current density [14, 27, 28, 32]. Interestingly, the apparent reduction in sodium current density associated with the A306del variant was statistically insignificant. In our electrophysiology experiments, the GPD1L-A306del was further associated with a negative shift in steady-state activation and a positive shift in steady-state of inactivation, alterations not typical for the cellular “loss-of-function” phenotype, typically found in BrS. These results may explain the lack of a BrS phenotype associated with A306del alone, as presented by individual I.2. indicating that the solely expression of the GPD1L variant is not responsible for the BrS disease. Nevertheless, although GPD1L-A306del induced changes of the biophysical properties of sodium channels failed to fully elucidate the pathomechanism and to explain all phenotypical differences within the family, they clearly show that GPD1L-A306del as well as GPD1L-WT influence Nav1.5 function. These effects may, eventually, intensify repolarisation heterogeneities in specified cardiac regions and influence the sodium window current. The latter, even if increased, is probably very small and therefore is not expected to remarkably alter sodium channel availability. In sum, the GPD1L-A306del variant adds to the arrhythmogenic potential of the heterozygous SCN5A-G1661R “loss-of-function” variant in the index patient. Such additive effects could markedly increase arrhythmogenicity in vivo [36, 37]. Our study is the first that functionally assesses the combination of a SCN5A and a GPD1L variant carried by the same individual [34]. Although not fully conclusive and limited by measurements in a heterologous expression system, our data are in line with the current view that arrhythmic BrS, in many cases, is not induced by a single genetic cause, but more likely determined by different genetic modifiers and other factors (sex, age, epigenetic regulation) [8]. Consequently, panel-based genetic sequencing in families affected by BrS is a valuable tool to more adequately recognize individuals possibly at risk for adverse outcome carrying more than one variant in susceptibility genes.

## Consent

All patients included in this study gave written informed consent for clinical and genetic investigation according to the research protocol.

### Supplementary Information

Below is the link to the electronic supplementary material.

Supplementary file1 (DOCX 1157 KB)

## Data Availability

The datasets generated during and/or analysed during the current study are available from the corresponding author on reasonable request. Sequencing data were deposited into the ClinVar database under accession number SCV002029075 and VCV000201523.9.
