# Supplementary material for: GPD1L-A306del modifies sodium current in a family carrying the dysfunctional SCN5A-G1661R mutation associated with Brugada syndrome
Source: Pflugers Arch. 2023 Dec 1;476(2):229–42. doi: 10.1007/s00424-023-02882-0 (PMC10791828; doi:10.1007/s00424-023-02882-0)
Supplement: Supplementary file 1 — Supplementary file1 (DOCX 1157 KB) [file 424_2023_2882_MOESM1_ESM.docx]

GPD1L-A306del modifies sodium current in a family carrying the dysfunctional SCN5A-G1661R mutation associated with Brugada syndrome

Francesca Semino^1,2^, Fabrice F. Darche^1^, Claus Bruehl^2^, Michael Koenen^1,3^, Heyko Skladny^4^, Hugo A. Katus^1,5^, Norbert Frey^1,5^, Andreas Draguhn^2^, Patrick A. Schweizer^1,5^*

^1^Department of Cardiology, Medical University Hospital Heidelberg, Heidelberg, Germany.

^2^Institute of Physiology and Pathophysiology, Heidelberg University, Heidelberg, Germany.

^3^Department of Molecular Neurobiology, Max Planck Institute for Medical Research, Heidelberg, Germany.

^4^SYNLAB MVZ Humangenetik Mannheim GmbH, Mannheim, Germany.

^5^German Center for Cardiovascular Research (DZHK), Partner Site Heidelberg/Mannheim, Heidelberg, Germany.

*** Correspondence:**Patrick A. Schweizer
patrick.schweizer@med.uni-heidelberg.de


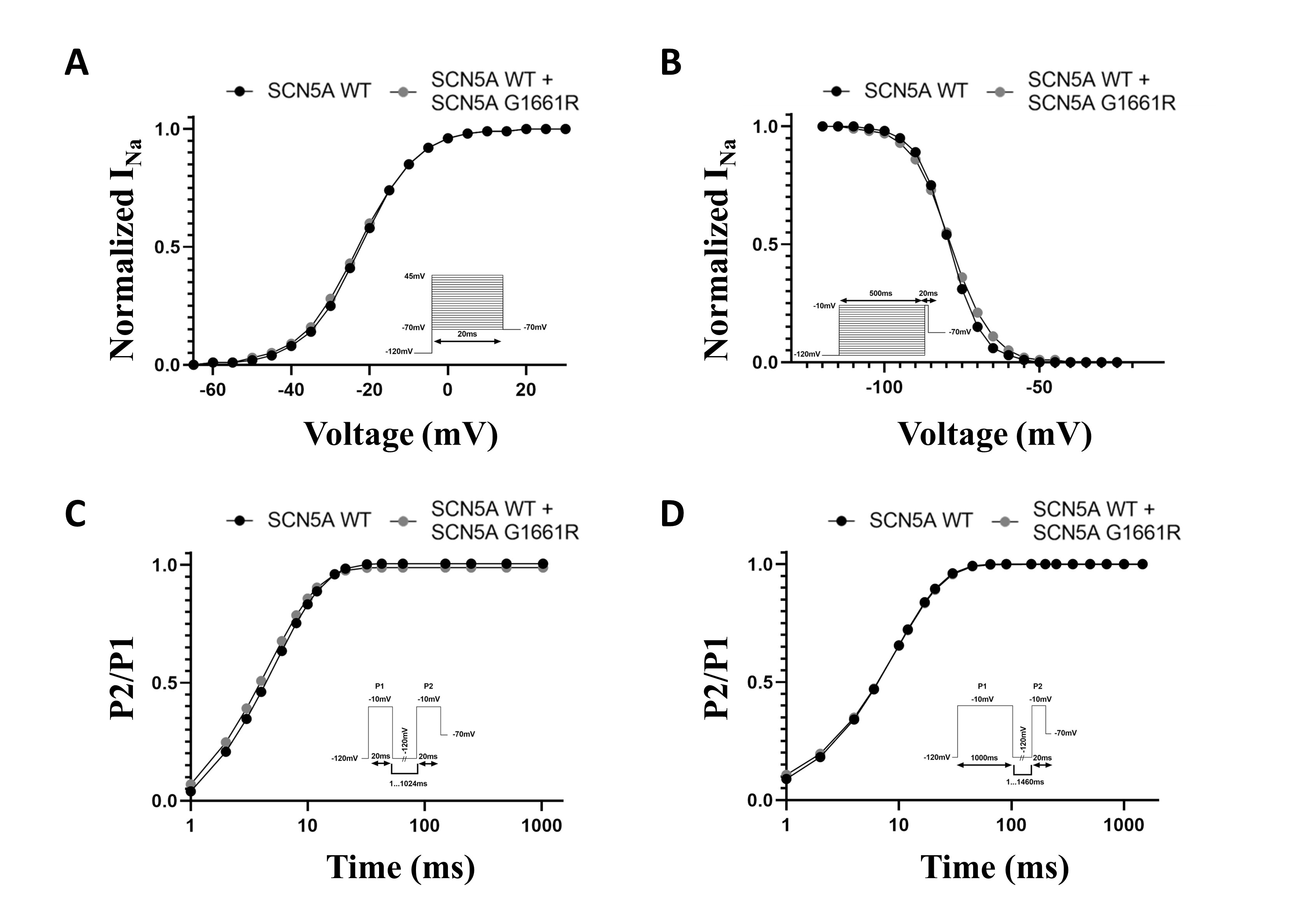


**Online Resource 1** SCN5A‑G1661R does not change the biophysical properties of the channels. (A-B) Voltage dependence of steady‑state activation (A) and inactivation (B) for WT (black circles) and heterozygous (grey circles) transfected *SCN5A* cells. The solid lines were determined using equations (1) and (2) with the averaged parameters. (C-D) Time dependence of recovery from fast (C) and intermediate (D) inactivation. The solid lines were determined using equation (3) with the averaged parameters


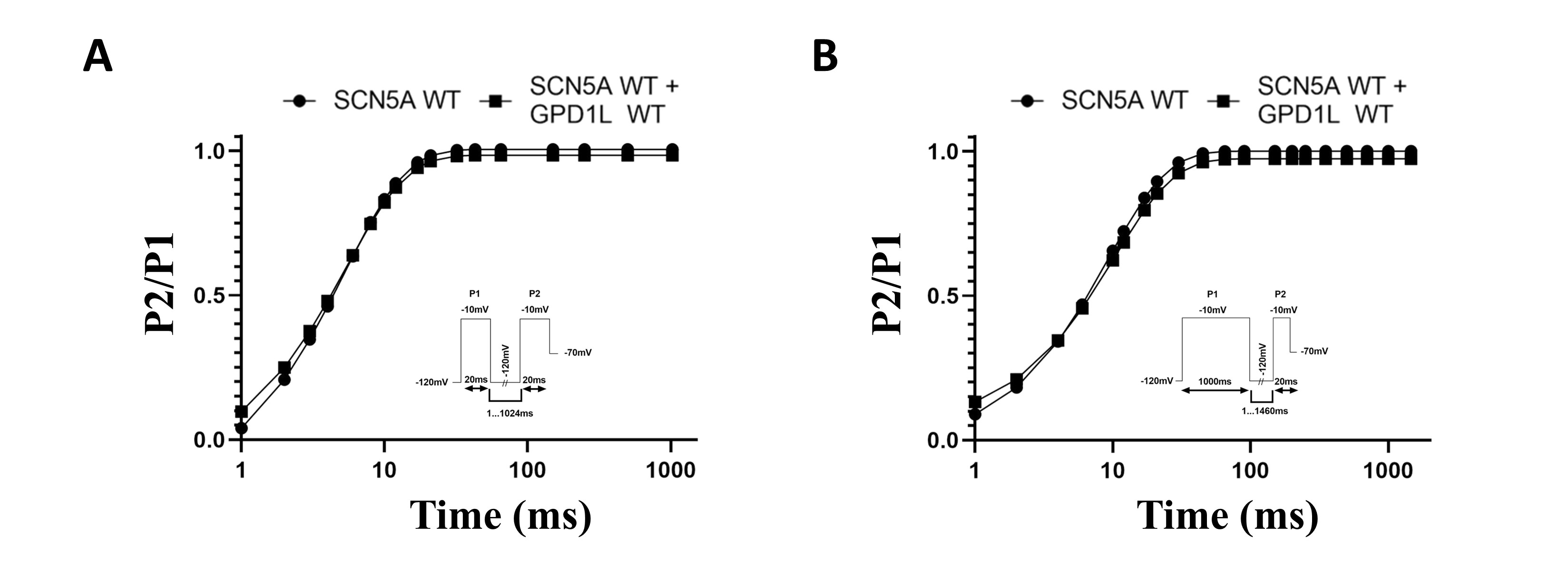


**Online Resource 2** GPD1L does not alter the recovery properties of the sodium channels. (A‑B) Time dependence of recovery from fast (A) and intermediate (B) inactivation for *SCN5A*‑WT (black circles) and SCN5A‑WT + GPD1L‑WT (black squares) transfected cells. The solid lines were determined using equation (3) with the averaged parameters


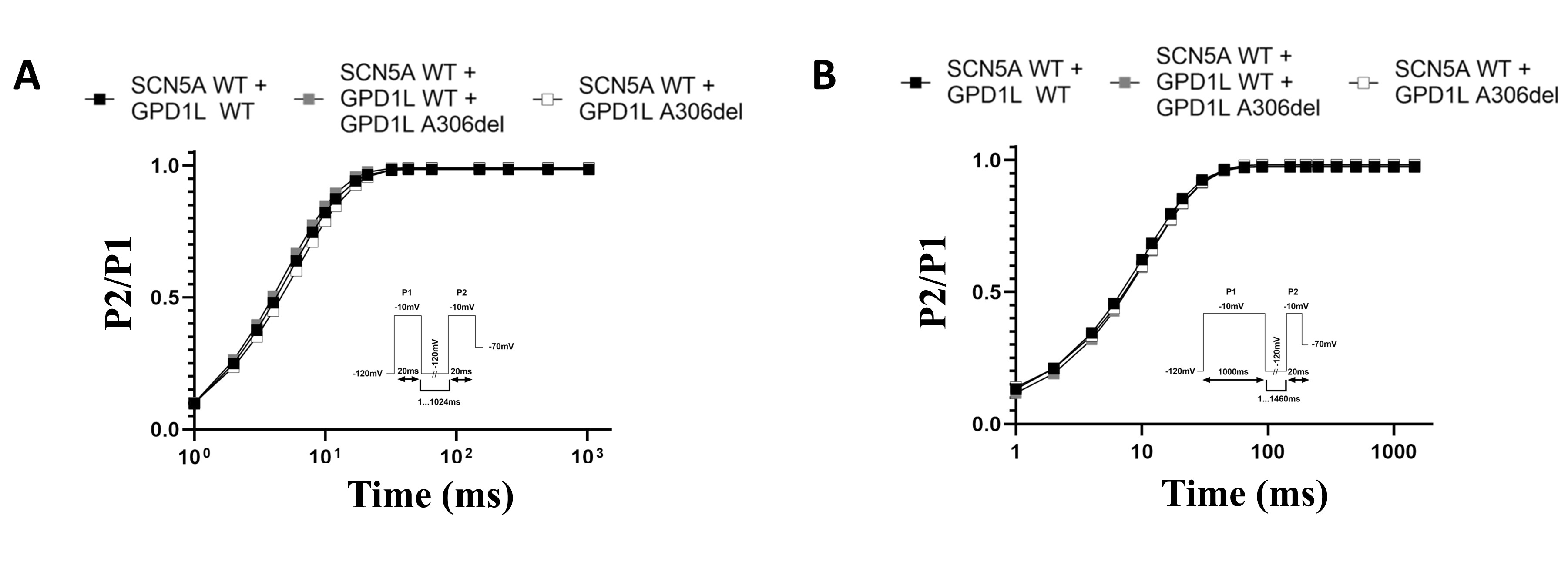


**Online Resource 3** GPD1L‑A306del does not alter the recovery properties of the sodium channels. (A-B) Time dependence of recovery from fast (A) and intermediate (B) inactivation for cells transfected with the WT *SCN5A* plasmid co-expressed either with the WT (black squares), the mutated (white squares) or both *GPD1L* plasmids (grey squares). The solid lines were determined using equation (3) with the averaged parameters


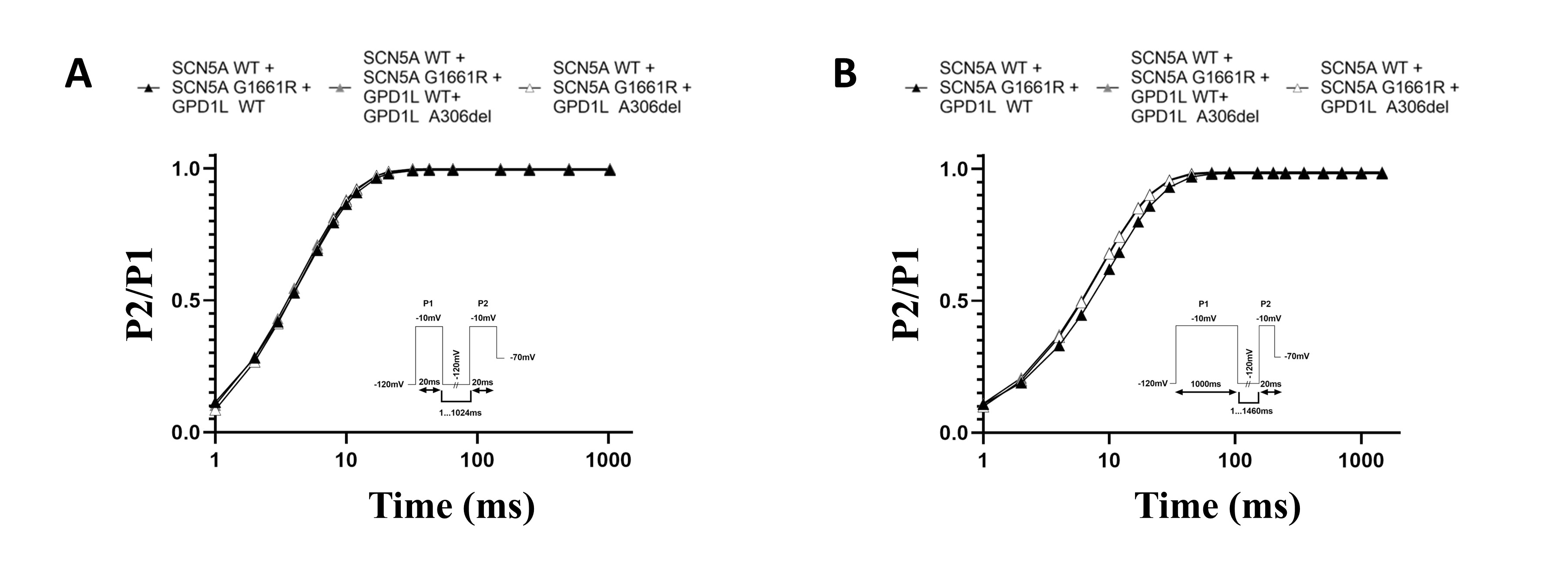


**Online Resource 4** SCN5A‑G1661R and GPD1L‑A306del co-expression do not alter the recovery properties of the sodium channels. (A-B) Time dependency of recovery from fast (A) and intermediate (B) inactivation of cells transfected with the WT and mutated *SCN5A* plasmid co-expressed either with the WT (black triangles), the mutated (white triangles) or both *GPD1L* plasmids (grey triangles). The solid lines were determined using equation (3) with the averaged parameters
